# Characterization of the complete mitochondrial genome and phylogenetic implications for *Eurydema maracandica* (Hemiptera: Pentatomidae)

**DOI:** 10.1080/23802359.2017.1365649

**Published:** 2017-08-17

**Authors:** Wanqing Zhao, Qing Zhao, Min Li, Jiufeng Wei, Xianhong Zhang, Hufang Zhang

**Affiliations:** a Department of Entomology, Shanxi Agricultural University, Taigu, China;; b Department of Biology, Taiyuan Normal University, Taiyuan, China;; c Department of Biology, Xinzhou Teachers University, Xinzhou, China

**Keywords:** *Eurydema maracandica*, Pentatomidae, Illumina sequencing, mitochondrial genome, phylogeny

## Abstract

The complete mitochondrial genome of *Eurydema maracandica* was sequenced and was 15,391 bp long with 76.82% A + T. There were 37 typical genes including 13 protein-coding genes/PCGs, 22 transfer RNA/tRNAs, and 2 ribosomal RNA/rRNAs. The 690 bp D-loop region was located between 12S rRNA and trnI-*Ile*. All PCGs started with ATN codons, except COI, ATP8, and ND1, and ended with TAA, except COI, COII, and ND5. Phylogenetic analyses indicated highly supported monophyly for each family, and Pentatomidae species formed a solid monophyletic group. *Eurydema maracandica* and *Eurydema gebleri* were clustered sibling clades, and the genus *Halyomorpha* was close to *Eurydema*.

*Eurydema maracandica* Oshanin, 1871 (Hemiptera: Heteroptera) is an agricultural pest of various brassicaceous vegetables (Yu et al. 2013; Zhao et al. 2017) that is prone to outbreaks, and an efficient control strategy for this insect is required. Mitochondrial genome analysis has proven to be a powerful tool for species diagnosis and is essential for comprehensive evolutionary studies (Cameron 2014). In this context, the complete mitochondrial genome of *E. maracandica* from Yining City (43.9873894674N, 81.6309202274E), Xinjiang Province, China, on 10 November 2011 was analyzed.

The complete mt genome, assembled from Illumina sequencing data and submitted to GenBank (accession: MF135553), was a 15,391 bp, typical double-stranded circular molecule with an asymmetric nucleotide composition (42.34% A, 13.23% C, 9.95% G, and 34.48% T). The order and orientation of 37 genes (including 13 PCGs, 22 tRNAs, and 2 rRNAs) were identical to that of most Pentatomidae species (Hua et al. 2008; Yuan et al. 2015). Of these, 14 (trnQ-*Gln*, trnC-*Cys*, trnY-*Tyr*, trnF-*Phe*, ND5, trnH-*His*, ND4, ND4L, trnP-*Pro*, ND1, trnL-*Leu* ^(CUN)^, trnV-*Val*, 16S rRNA, and 12S rRNA) were located on the light strand (L-strand); however, the other genes and the D-loop region were on the heavy strand (H-strand).

All PCGs shared the start codon ATN (ATA, *n* = 3; ATT, *n* = 3; ATG, *n* = 4) – except COI, ATP8, and ND1, which started with TTG – and terminated with TAA, except COI, COII, and ND5, which ended with a single T. Sixteen instances of intergenic regions (1–29 bp) and eight instances of overlapping regions (1–7 bp) were found. The 22 tRNAs ranged from 64 to 72 bp, and all had a typical cloverleaf structure except trnS-*Ser* ^(AGN)^ and trnV-*Val*, which lacked a dihydrouridine arm. The two rRNAs were 1281 bp (16S rRNA) and 811 bp (12S rRNA) long and were separated from each other by trnV-*Val*. The D-loop region was located between 12S rRNA and trnI-*Ile* with a total length of 690 bp.

Phylogenetic analyses of 13 superfamily Pentatomoidea species and three superfamily Coreoidea species were conducted using maximum-likelihood (ML) methods on the 13 PCGs (Figure 1). An ML tree was constructed using RAxML 7.0.3 (Stamatakis 2006) with the GTR + I + G model estimated by PartitionFinder v1.1.0 (Lanfear et al. 2012). Each family formed a monophyletic cluster with a high degree of bootstrap support, and each Pentatomidae genus was monophyletic. *Eurydema* was more closely related to *Halyomorpha*, and *E. maracandica* and *E. gebleri* were sibling clusters. The complete mitogenome of *E. maracandica* provides important molecular and evolutionary evidence for *Eurydema* and Pentatomidae.

**Figure 1.**
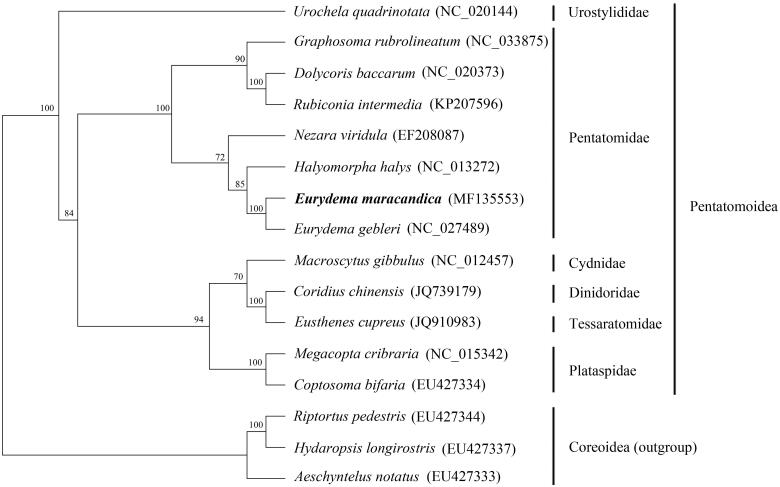
Phylogenetic relationship of *E. maracandica* within Pentatomoidea inferred from 13 PCGs. Numbers at the nodes are bootstrap values.
